# Supplementary material for: Pharmacists’ Knowledge, Attitude and Practice Regarding the Dispensing of Antibiotics without Prescription in Tanzania: An Explorative Cross-Sectional Study
Source: Pharmacy (Basel). 2020 Dec 13;8(4):238. doi: 10.3390/pharmacy8040238 (PMC7768476; doi:10.3390/pharmacy8040238)
Supplement: Supplementary file 1 [file pharmacy-08-00238-s001.pdf]

Supplementary Material File S1: Online questionnaire

MUHIMBILI UNIVERSITY OF HEALTH AND ALLIED SCIENCES

SCHOOL OF PHARMACY

DEPARTMENT OF PHARMACEUTICAL MICROBIOLOGY

DISPENSING OF ANTIBIOTICS

Dear Pharmacist,

My name is POYONGO BARAKA a fouth year student pursuing a bachelor degree in pharmacy at MUHAS. I am currently conducting a survey on *Pharmacists' knowledge attitude and practice regarding dispensing of antibiotics in* Tanzania,**for my final year reseach project. This survey is for academic purpose, please feel free to respond to the questions because information that identify a person like his/her name and name of his/her pharmacy are not asked. All the responses will be confidential where the provided information will be coded and no participant will be identified in either way. Your participation in this survey is voluntary and you can withdrawal whenever you feel uncomfortable in answering the questions.**

The findings from this survey on one hand will be useful to the universities to assess the current level of knowledge about antibiotic resistance to Pharmacists and determine the need for curriculum change. On the other hand, the reasons for antibiotics dispensing without prescription will be revealed and recommendation drew to advise the Government on strategies for combating the ever growing problem of antimicrobial resistance and consequent failure to effectively treat bacterial infections.

AFTER FINISHING TO RESPOND TO THE QUESTIONS, PLEASE REMEMBER TO CLICK SUBMIT SO THAT YOUR RESPONSE CAN BE RECORDED.

Please spare few minutes to respond to the questions and thank you for your time.

Please read the survey invitation in the attached document

Attachment: SURVEY INVITATION LETTER.pdf (0.38 MB)

This is a voluntary survey, if you do not wish to respond to the questions please feel free to exit this survey now. Do you wish to participate in this survey? ○ Yes ○ No reset

* must provide value

Sex
* must provide value

- ◯ Male
- ◯ Female

reset

Age
* must provide value

[ ]
This is confidential -not linked to a person

Maximum education level attained
* must provide value

- ◯ Bachelor
- ◯ Masters
- ◯ PhD

reset

Country from which you graduated your bachelor of pharmacy
* must provide value

- ◯ Tanzania
- ◯ Kenya
- ◯ Uganda
- ◯ Other

reset

Do you work as community pharmacist in any of the registered community pharmacy ?
* must provide value

- ◉ Yes
- ◯ No

reset

What is your job status in that community pharmacy?
* must provide value

- ◯ Owner
- ◯ Dispensing pharmacist
- ◯ Both Owner and dispensing pharmacist

reset

In which region is your working pharmacy located?
* must provide value

[ ]
Type some characters of the region

By comparing the number of patients who come to the pharmacy with prescription and those who come without prescription, what is the percent of patients who come to your pharmacy asking for antibiotics without prescription per day?
* must provide value

0 50 100
Change the slider above to set a response
reset
Select 1-100

For how long have you been practising your profession as pharmacist in either community pharmacy or in other health facility/facilities (in years)?
* must provide value

[ ]
1 - if you just graduated/from internship

SECTION II

DONT FORGET TO CLICK SUBMIT AT THE BOTTOM OF THIS PAGE

Dispensing antibiotics without prescription is a legal practice in Tanzania.
* must provide value

- Yes
- No
- Don't know

reset

Dispensing antibiotics without prescription is a common practice among pharmacists in Tanzania
* must provide value

- Yes
- No
- Don't know

reset

Do you think there is any problem if you dispense antibiotic without prescription?
* must provide value

- Yes
- No
- Don't know

reset

Dispensing antibiotics without prescription is contributing to the inappropriate use of antibiotics by patients
* must provide value

- Yes
- No
- Don't know

reset

Dispensing antibiotics without prescription is contributing to development of Antimicrobial Resistance.
* must provide value

- Yes
- No
- Don't know

reset

Antibiotic resistance has become a public health issue
* must provide value

- Yes
- No
- In other countries except for Tanzania
- Don't know

In Tanzania, a Pharmacist can be penalized for dispensing antibiotics without a prescription.
* must provide value

- Yes
- No
- Don't know

reset

Pharmacist should stop dispensing antibiotics without prescription
* must provide value

- Yes
- No
- Don't know

reset

I encourage patients to consult the physician and get a prescription
* must provide value

- Always
- Sometimes
- Never

reset

SECTION III

DONT FORGET TO CLICK SUBMIT AT THE BOTTOM OF THIS PAGE

Which factor(s) do you think force a patient to go to the pharmacy without prescription asking for an antibiotic when he/she is sick.

* must provide value

- ☐ Patient doesn't want to see a doctor unless the infection is serious
- ☐ Patient doesn't afford the cost for consultation and laboratory tests
- ☐ Fear to use many hours to complete the medical process for a prescription when attending hospital
- ☐ Lack of Insurance cover
- ☐ Other

Pharmacists have good knowledge of antibiotics hence sometimes they can dispense without prescription after critical evaluation of patients' sickness

* must provide value

- ○ Yes
- ○ No

reset

Do you think a pharmacist can deny antibiotics to his/her sicking relatives (father, mother, brother, sister and others) who present to pharmacy without prescription?

* must provide value

- ○ Yes
- ○ No

reset

Do you think pharmacist dispense antibiotics without prescription due to lack of awareness about rules and regulations regarding dispensing of antibiotics?

* must provide value

- ○ Yes
- ○ No

reset

If there could be a regular inspections of dispensing practices in the pharmacy by regulatory body, do you think the pharmacists will still dispense antibiotics without prescription?

* must provide value

- ○ Yes
- ○ No

reset

If there could be a regular inspections of dispensing practices in the pharmacy by regulatory body, do you think the pharmacists will still dispense antibiotics without prescription?

* must provide value

- Yes
- No

reset

Based on the nature of pharmacy business, choose one factor that mostly influence a pharmacist to dispense antibiotics without prescription.

* must provide value

- Fear of losing customers
- Increased sales and profit pressure from the owner
- When patients feel that they need an antibiotic, if not dispensed, they will try to obtain it from another pharmacy
- other factor

reset

SECTION IV

DONT FORGET TO CLICK SUBMIT AT THE BOTTOM OF THIS PAGE

Between broad spectrum antibiotic and narrow spectrum antibiotic, which one you think are commonly dispensed without prescription?

* must provide value

- Narrow spectrum
- Broad spectrum
- Both narrow and broad spectrum

reset

Which class of antibiotic you will READILY AND EASILY dispense to a patient who has no prescription suffering from suspected bacterial infection? Choose from the list.

* must provide value

- Cephalosporins eg ceftriaxone, Cephalexin,Cefaclor
- Penicillins, eg Amoxicillin, Ampicillin, Ampiclox
- Floroquinolones eg Ciprofloxacin, Norfloxacin
- Tetracyclines, eg Doxycycline, Tetracycline
- Macrolides, eg Azithromycin, Erythromycin
- Aminoglycosides,eg Gentamicin,Neomycin
- Sulphonamides, eg Sulfamethoxazole

What is the common dosage form of antibiotics dispensed without prescription? CHOOSE TWO from the list.
* must provide value

- Oral
- Ear drops
- Eye drops
- Topical
- Injection
- Intravascular

Common medical conditions for which antibiotics are dispensed without prescription (CHOOSE TWO from the list given.)
* must provide value

- Toothache
- Cold and Flue
- Diarrhea
- Urinary tract infections (UTI)
- Cough
- Rhinitis
- Sexual transmitted Infections (STI)
- Others

SECTION V

DONT FORGET TO CLICK SUBMIT AT THE BOTTOM OF THIS PAGE

Do you dispense antibiotics to a person who has no prescription?
* must provide value

- Always
- Sometimes
- Never

When was your last time to dispense antibiotic/s without prescription?
* must provide value

Will you stop this practice in future?
* must provide value

- Yes
- No

Please, explain in briefly why you think so.
* must provide value

When dispensing antibiotics without prescription, I ask patients about drug allergies.
* must provide value

- Always
- Sometimes
- Never

When dispensing antibiotics without prescription, I warn patients about the potential side effects of the medicines
* must provide value

- Always
- Sometimes
- Never

When dispensing antibiotics without prescription, I educate patients about the importance of adherence and completing the full course of antibiotics
* must provide value

- Always
- Sometimes
- Never

When dispensing antibiotics without prescription, I educate patients about the importance of adherence and completing the full course of antibiotics

* must provide value

- Always
- Sometimes
- Never

When dispensing antibiotics without prescription, I ask patients if they are taking any other medication for the same complaint

* must provide value

- Always
- Sometimes
- Never

I don't dispense antibiotics without prescription for children

* must provide value

- Always
- Sometimes
- Never

Do you dispense antibiotic to a pregnant woman who has no prescription?

* must provide value

- Always
- Sometimes
- Never

Please click the word 'Submit' shown bellow so as your response may be recorded.

Submit
